# Convalescent plasma therapy: a promising coronavirus disease 2019 treatment strategy

**DOI:** 10.1098/rsob.200174

**Published:** 2020-09-09

**Authors:** Ravikant Piyush, Keshav Rajarshi, Rajni Khan, Shashikant Ray

**Affiliations:** 1 School of Biotechnology, Madurai Kamaraj University, Madurai, Tamil Nadu 625021, India; 2 School of Community Science and Technology (SOCSAT), Indian Institute of Engineering Science and Technology (IIEST), Shibpur, Howrah, West Bengal 711103, India; 3 Motihari College of Engineering, Bariyarpur, Motihari, NH 28A, Furshatpur, Motihari, Bihar 845401, India; 4 Department of Biotechnology, Mahatma Gandhi Central University, Motihari 845401, India

**Keywords:** COVID-19, SARS-CoV-2, plasma therapy, viraemia, chloroquine

## Abstract

The world is passing through a very difficult phase due to the coronavirus disease 2019 (COVID-19) pandemic, which has disrupted almost all spheres of life. Globally, according to the latest World Health Organization report (10 August 2020), COVID-19 has affected nearly 20 million lives, causing 728 013 deaths. Due to the lack of specific therapeutic drugs and vaccines, the outbreak of disease has spawned a corpus of contagious infection all over the world, day by day, without control. As the severe acute respiratory syndrome coronavirus 2 (SARS-CoV-2) has a very rapid infection rate, it is essential to develop a novel ameliorative and curative strategy as quickly as possible. Convalescent plasma (CP) therapy is a type of adaptive immunity that has already been found to be effective in confronting several infectious diseases from the last two decades. For example, CP therapy was used in the treatment of viral-induced diseases like SARS-CoV epidemics, Middle East respiratory syndrome coronavirus (MERS-CoV) pandemics, Ebola epidemics and H1N1 pandemic. In this review, we have mainly focused on the therapeutic role of CP therapy and its neutralizing effect to fight against the COVID-19 outbreak.

## 1. Introduction

The first case of SARS-CoV-2 was reported in Wuhan, Hubei province of China, in November 2019 [1]. The disease remained asymptomatic in its initial stages of infection, and earlier, it was confused with normal seasonal flu due to the mild symptoms. As the severity of the disease increased in the following days, and the number of people suffering from respiratory tract infections escalated, soon it became a matter of global concern [2]. As of now, around 210 countries and more than 20 million people across the globe are affected by this virus [3]. World Health Organization (WHO) described this coronavirus as a novel virus on 1 January 2020, declaring the outbreak as an international emergency [4]. Later, the phylogenetic analysis of the novel viral genome revealed that it shared 89.1% similarity to the severe acute respiratory syndrome (SARS) coronaviruses, and consists of 29 903 nucleotides which are enveloped with positive-sense single-stranded RNA [5,6]. WHO abbreviated the novel coronavirus as SARS-CoV-2, and the new disease coronavirus disease 2019 as COVID-19 [4].

Coronaviruses (CoVs) belong to the family of Coronaviridae, subfamily Orthocoronavirinae, order Nidovirales, and the subfamily includes α-coronavirus, β-coronavirus, γ-coronavirus and δ-coronavirus [7]. In the past few decades, several findings reported these viruses crossing the species barrier, consequently infecting humans as well. Many human or animal CoVs have been seen to have originated in bats [8–10]. The occurrence of SARS in 2002 and MERS in 2012 has made us aware of the extreme transmissibility and lethality of the coronavirus family in humans [8]. SARS-CoV and MERS-CoV (Middle East respiratory syndrome coronavirus) both belong to the β-coronavirus subfamily, like the novel SARS-CoV-2 [11]. The genome size of the novel SARS-CoV-2 was found to be 30 kb long [6,12]. The microscopic images of CoV show that the virions consist of nucleocapsid core covered by an envelope, which is made up of three membrane proteins, such as membrane (M), spike (S) and envelope (E) proteins. These membranous proteins are highly conserved in the CoVs. The genetic material (i.e. RNA) is packaged by the nucleocapsid (N) and other accessory proteins. The non-structural protein-encoding genes are also present at the genome's 5′-end [5,13].

Until now, no specific treatment has been discovered to be completely effective against the virus. The scientific community is still struggling to develop a potent vaccine to prevent infection [14]. Presently, the management of SARS-CoV-2-infected patients basically emphasizes the use of medicines like hydroxychloroquinone, chloroquinone, azithromycin and ivermectin, along with vitamin B and vitamin C supplements; supportive care includes oxygenation, management of fluid and ventilation [15–17]. As a part of critical COVID-19 management, atomization inhalation of interferon, along with the systematic administration of low doses of corticosteroids, has also been recommended [15].

Passive immunotherapeutic strategies like convalescent plasma (CP) therapy have been used by several countries to treat patients suffering from severe SARS-CoV-2 infection [18,19]. CP has proved its effectiveness in neutralizing viruses like SARS-CoV, MERS-CoV, Ebola and H1N1. So, in this pandemic situation of COVID-19, globally, CP therapy has been explored for an effective line of treatment [20,21].

### 1.1. SARS-CoV-2 pathogenesis and immune response

SARS-CoV-2 was found to enter the host cell by using their S protein [22]. The S protein of SARS-CoV-2 binds with the human angiotensin-converting enzyme 2 (hACE2) receptors present on the epithelial surface of several organs like lungs, heart, kidney, brain and small intestine, and thereby several organs are found to be infected in the course of illness [23,24]. The pathogenesis of SARS-CoV-2 is highly complex due to the binding of S protein to the hACE2 receptor present on the several organs. It has also been reported that SARS-CoV-2 damages several organs, including the alveoli of the lungs [24]. The crystal structure of S protein reveals that it has two main globular domains: S1 and S2 [22]. The attachment of the S protein to the hACE2 receptor is facilitated by the receptor-binding domain present in the S1 subunit of S glycoprotein [25,26]. The host cell enzymes like TMPRSS211, a serine protease, and lysosomal protease cathepsins facilitate the invasion of the virus inside the host cell by cleaving the S protein at the junction of S1–S2, resulting in the conformational changes in the S2 domain, which is necessary for fusion of the virus to the host cell membrane [26,27]. SARS-CoV-2 also damages several immune cells, which leads to the activation of nuclear factor kappa-light-chain-enhancer of activated B cells (NF-κB) and signal transducer and activator of transcription 3 (STAT3). The hyperactivation mechanism of NF-κB by STAT3 leads to the activation of the interleukin-6 amplifier (IL-6 Amp), resulting in multiple inflammatory and autoimmune diseases [28].

Interestingly, the exposure of SARS-CoV-2 does not infect the entire population, and not all infected persons acquire severe respiratory illness. This differential infection and illness may depend upon the immune response of individuals. Based on the differential response, the SARS-CoV-2 infection is clinically categorized into mainly two stages [16]: non-severe and severe.

#### 1.1.1. Non-severe stage

To prevent infection at an initial stage, a specific adaptive immune response is needed to stop the disease progression. So, several immune booster food supplements and vitamins have been recommended to enhance immunity [16]. The disease progression varies depending on the genetic composition of the individuals. Due to the differences in the genetic background, the immune system of individuals responds differently to the infection [29].

#### 1.1.2. Severe stage

This is also known as a severe respiratory symptomatic stage with high viral load. In this stage, the virus is able to impair the immune response, which leads to the multiplication of the virus, which further results in enormous obliteration of the affected tissues, especially the organs which have a high level of expression of hACE2 receptors [30]. Therefore, the virus damages the tissues of kidneys and lungs more prominently [16,30]. Proinflammatory granulocytes and macrophages mediate an innate inflammation in the lungs, which are induced by the damaged cells, and therefore even a strong immune system and good health are not very helpful in the severe stage [16]. In the severe stage, the main cause of the fatal respiratory ailments is inflammation in the lungs [31].

## 2. The concept of passive antibody therapy

In order to treat or prevent infection of particular diseases, the susceptible individual is administered with antibodies against the required antigen [32,33]. The active immune response develops against any diseases either due to infection from a pathogen or due to the administration of specific vaccine against the disease that triggers the immune response. The active immune response takes time for the generation of antibodies, and the time differs depending on the immune system of the recipients [34]. Therefore, passive antibody administration is the only technique to provide immediate immunity to vulnerable individuals in a short span of time. The passive antibody therapeutic strategy dates back to the late nineteenth century. During the 1890s, in order to treat certain diseases that were transmissible or infectious, passive antibody therapy was used [35,36]. Emil von Behring discovered serum therapy to treat diphtheria, which was recognized for the Nobel Prize in Physiology or Medicine in 1901 [37]. Furthermore, Behring and Shibasaburo Kitasato reported that tetanus in rabbits could be prevented by immunizing the rabbit serum with tetanus toxin priorly [38].

The basic principle involved in passive antibody therapy is that it is more worthwhile when used for prevention or prophylaxis rather than for the cure or treatment of the disease. When the antibodies are used for therapy, they need to be administered soon after the commencement of the symptoms so that their efficacy is maintained [32]. The reason for time-related variation in the efficiency of passive antibodies is yet to be discovered, but this could mean that the passive antibodies neutralize the initial inoculum, which is expected to be very much smaller than that of the prevailing disease [39]. According to another interpretation, the antibody works via modification of the inflammatory response, which is achieved conveniently during the previous/initial immune response, probably an asymptomatic stage [40]. The expected mechanism of action through which the passive antibody therapy would regulate protection in the case of SARS-CoV-2 involves the neutralization of the virus, though several other mechanisms may also be possible, including antibody-mediated cellular cytotoxicity or phagocytosis [41]. The potential antibody sources for SARS-CoV-2 are human convalescent sera from the individuals who have recuperated from COVID-19, and preparations made in certain animal hosts like genetically engineered cows producing human antibodies or mAbs (monoclonal antibodies) [42]. The number of potential donors will rise as more people go down with COVID-19 and recover.

### 2.1. Why convalescent plasma therapy?

The idea of CP infusion was proposed in the nineteenth century when it showed immunity against diphtheria [41]. The existing antibodies in the blood derived from animals who were purposely immunized with a non-lethal dosage of toxins were administered to animals suffering from an ongoing infection, thus providing them with passive immunity [43,44]. It was then realized that the immune plasma provided passive immunomodulatory properties, along with pathogen neutralization, which enabled the recipient to avoid the inflammatory cascade triggered by various infectious agents [44,45]. The purification and concentration of immunoglobulins from recovered patients and healthy donors in the 1950s provided an alternative to treat severely infectious ailments as well as compromised immune conditions including allergies, autoimmune diseases and primary immunodeficiencies [43,46,47].

Be it the current pandemic caused by SARS-CoV-2 or Spanish influenza caused by H1N1, it has been suggested that the implementation of CP remarkably reduces the case fatality rate [48]. Moreover, the use of CP in other CoVs like SARS-CoV resulted in a reduction in the number of days that a severely sick patient was supposed to spend in the hospital [49,50]. Keeping in view the usage of mechanized ventilation systems during the avian influenza A (H5N1) and the 2009 pandemic of influenza A (H1N1), the implementation of CP therapy decreased the duration of invasive and obtrusive ventilation [51,52]. Presently, the administration of CP to COVID-19 patients has exhibited improvement in their clinical condition and a reduction of the viral load [53,54].

Intravenous immunoglobulins (IVIg), monoclonal or polyclonal antibodies, and various other convalescent blood products have been developed to deal with infectious indispositions [55]. Despite this fact, they may not yield appropriate infection control and are quite difficult and expensive to produce. Therefore, due to the lack of effective vaccines and medications, CP therapy has been broadly implemented during various outbreaks as the first therapeutic alternative and sometimes as an experimental treatment or last resort [45].

## 3. The strategy of convalescent plasma therapy

CP therapy has been used to prevent and treat several infectious diseases for more than one century and is one of the classic adaptive immunotherapies [54]. CP's therapeutic strategy has been successfully implemented in treating SARS, MERS and H1N1 with reasonable efficiency and safety in the past two decades [56–59]. Results obtained from the meta-analysis of 32 different studies focusing on severe influenza infections and SARS-CoV infections statistically suggested that a significant reduction was observed in the chances of fatalities and rates of mortality due to CP therapy [60]. To enhance the efficiency of antibody therapies to neutralize the virus, collection and utilization of hyperimmune IgG antibodies from individuals (subjects) who were previously infected by SARS-CoV-2, but now have recovered from COVID-19, is preferred as they have enhanced production and hence an increased amount of the antibodies [61]. As several strains of the virus exist, and their spread is non-uniform (i.e. the pattern of spread varies in different cities and countries), the potential plasma donors should belong to the same geographical area as the recipients. Cytotoxicity and phagocytosis can be modulated via passive antibody therapy, and in combination with antiviral drugs, viral neutralization can be achieved [32]. The duration of therapy and the amount of antibodies in the convalescent serum mainly depend on the severity of COVID-19 and the viral load [62]. For treating or preventing the initial symptoms of COVID-19, virus-neutralizing antibodies in small amounts are considered to be effective. Individuals having chronic underlying diseases, healthy individuals who have been in contact with infected patients and healthcare workers are administered with the serum, and hence, induced passive immunity may last for a few weeks or months [32].

### 3.1. Convalescent plasma therapy: composition of plasma and its extraction

The composition of CP varies and encompasses several varieties of blood-derived elements. The plasma comprises a combination of organic compounds, water and inorganic salts, and consists of more than a thousand different kinds of enzymes and proteins like albumin, complement factors, coagulation factors, antithrombotic factors and immunoglobulins [63]. It is also assumed that plasma obtained from healthy donors imparts immunomodulatory effects through the infusion of antibodies and anti-inflammatory cytokines, which impedes the action of autoantibodies and inflammatory cytokines [64].

A standard pre-donation assessment of the convalescent donors is done as it is essential to adhere to the current plasma donation regulations [65]. Presently, convalescent donors between the age of 18 and 65 are regarded as subjects. They should be COVID-19 negative after 14 days and should not display any infective symptoms. These tests must be conducted again after 48 h and at the time of donation [54,65]. Plasma donors belong to areas with prevailing diseases (i.e. the endemic areas should be precluded). Apart from medical examinations and molecular evaluations, it is also very important to identify the emotional conditions of the donor in order to ensure they do not feel exploited during the donation [66]. The recommended procedure for plasma extraction is apheresis, which involves continuous centrifugation of blood obtained from the donor in order to allow a selective collection plasma. The efficacy of this technique ranges from 400 to 800 ml from a single apheresis donation. For its use in future transfusions, the plasma is stored in units of 200 or 250 ml and is frozen within 24 h of collection to retain its potency [67]. Conduction of medical examinations and clinical evaluation for hepatitis B and C, human immunodeficiency virus (HIV), human T-cell lymphotropic virus types 1 and 2, *Trypanosoma cruzi* (if residing in an endemic area) and syphilis are essential as high-quality standards are required in CP production, and it should be free from infections of any sort [65,68]. Therefore, in order to assure the safety of the recipients, it is compulsory to perform a nucleic acid test for HIV and hepatitis viruses [69]. Neutralization of pathogens with UV light exposure or with the aid of riboflavin are some other suggested procedures to improve safety in the production of CP [70].

## 4. Antiviral mechanism of convalescent plasma therapy against SARS-CoV-2

Neutralizing antibodies play a crucial role in the elimination of viral infection and are believed to be essential in prevention from viral illnesses [71]. Neutralizing antibodies that prevent these infections are provided by passive immunity, impelled by CP. The efficiency of this therapy is primarily influenced by the amount of neutralizing antibodies in the plasma obtained from the recuperated donors [72,73]. In the case of MERS and SARS-CoV, it was found that the neutralizing antibodies inhibit the viral amplification by binding to the N-terminal domain of S1, receptor-binding domain (RBD) of S1 and S2 domains of S protein, and thereby help in controlling the infection [74]. Furthermore, CP administration is also known to enhance antibody-dependent cellular cytotoxicity, among other effects [67].

**Figure 1.**
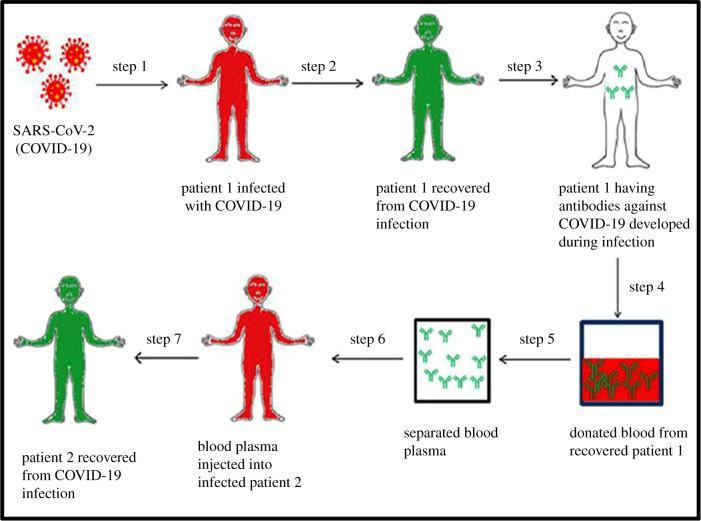
Schematic of plasma therapy. When the COVID-19-infected individuals recover, their blood plasma contains antibodies against the COVID-19 causing SARS-CoV-2 virus. The recovered individuals donate their blood, from which the plasma containing the required antibodies is extracted. This plasma is then administered to the infected individual(s) via transfusion.

After SARS-CoV infection, IgG antibodies are produced in response to the nucleoprotein of the virus, which is generally detected on the seroconversion on the 14th day or on the 4th day after the beginning of disease [75]. Even after 2 years of SARS-CoV infection, around 89% of the convalesced patients displayed neutralizing antibodies and IgG-specific antibodies [76]. A study conducted using biolayer interferometry binding and ELISA suggested that a CR3022, which is a SARS-CoV-specific antibody, binds with the RBD of S protein of SARS-CoV-2, without competing with the hACE2. The RBD of S protein of both SARS-CoV-2 and SARS-CoV shows a high variation in the amino acid at their C-terminal region. However, such variations did not affect the capability to engage the hACE2 receptor but had a crucial impact on the cross-reactivity of neutralizing antibodies [77]. Apart from the neutralizing antibodies in the plasma, other safeguarding antibodies like IgM, IgG and some non-neutralizing antibodies were found to interact with the virus by binding to it, which may contribute towards an enhanced recovery rate or may promote prophylaxis [67], but it did not intervene in the viral replication. Figure 1 briefly describes the mechanism of CP therapy.

## 5. Potential risks and challenges associated with CP therapy

In various studies, it has been shown that viral infections in the blood, such as SARS-CoV infection, are at their maximum during the first week of the infection [56]. After the onset of symptoms in the second week, lethal cytokine storms are triggered due to the development of immune response by the patients, which could be reduced by CP infusions [58,56 78]. However, there may be some concealed risks associated with CP therapy, like aggravated hyperimmune attacks [55]. The risks associated with the administration of CP have been categorized as known and theoretical risks [32]. The known risks include reactions against the plasma constituents and unintended infections induced by several infectious agents that might be present in the serum [32]. The theoretical risks include phenomena like antibody-dependent enhancement of infection, in which the severity of a viral disease is enhanced in the presence of specific antibodies [32]. During transfusion-related events, the most prevalent adverse effects of CP therapy can be observed, including fevers, anaphylactic reactions, chills, transfusion-associated circulatory overload, haemolysis and transfusion-related acute lung injuries (TRALIs) [79]. At the same time, HIV, syphilis, hepatitis B and C, and various other transfusion-mediated infections should also not be neglected [80–82]. In the case of developing and populous countries like India, if the disease spreads at a rapid rate and the infected persons exceed the recovered patients, there could be a shortage of available plasma for CP therapy. Besides, another potential challenge to this therapy could be the availability of plasma for the rare blood group-type patients [83].

## 6. Accomplishments using CP therapy

Due to the absence of any specified treatment for COVID-19, researchers are still searching for potential therapeutic agents and an effective line of treatment to contain the disease. The implementation of CP therapy against COVID-19 was checked based on its past trials and the successful treatment of several viral-mediated epidemics and pandemics [84]. To date, several studies have reported the efficacy of CP therapy in curing patients with SARS-CoV-2 infection [54,85,86]. Table 1 precisely enlists the CP administration in previously encountered CoV diseases, along with COVID-19.

**Table 1.** CP dosage during previously encountered coronavirus outbreaks along with the novel coronavirus.

disease and the causative agent	CP dose (volume)	antibody titre	summarized findings	references
SARS caused by SARS-CoV	279 ± 127 (160–640) ml 200 ml 500 ml 2 units of 250 ml each (total 500 ml)	NA NA IgG: >640 NA	—overall, 80 patients received CP; ten patients died —CP at approximately 14 (7–30) days following the onset of symptoms —good clinical outcome; by day 22, 33 patients were discharged from the hospital —improved outcome with early administration of CP —no adverse events	[49,67,87–89]
MERS caused by MERS-CoV	four transfusions of CP to three patients; volumes not stated 2 units (250–350 ml/unit) 250 ml	1:40 to 1:80 NA NA	—all three patients survived, questionable benefits —feasibility study to evaluate the ratio of convalescent donors having antibodies against MERS-CoV —case report of one patient —TRALI observed	[67,90–92]
COVID-19 caused by SARS-CoV-2	200 ml two consecutive transfusions of 200–250 ml (400 ml total)	neutralizing antibody titre: >1:640 ELISA anti-SARS-CoV-2—antibody titre: >1:1000 neutralizing antibody titre: >1:40	—uncontrolled, 10 severely ill patients, CP at 16.5 (11.0–19.3) days; recovery of all patients; no significant adverse effect —uncontrolled five critically ill cases, CP at 10–22 days after admission; recovery of all patients	[53,93]

One study included 10 severely affected patients who were confirmed positive in a real-time viral RNA test. The patients were administered with a single dose of 200 ml CP, which was extracted from recently recuperated individuals. The virus-neutralizing antibody titres in the extracted CP were above 1:640 and were delivered to the patients as additional support along with other antiviral agents. Within 3 days of administration of CP in the patients, oxyhaemoglobin saturation was enhanced, and clinical symptoms remarkably improved. The lymphocyte count, which was 0.65 × 10^9^ per litre prior to CP administration, escalated to 0.76 × 10^9^ per litre. A reduction in the concentration of C-reactive protein from 55.98 to 18.3 mg l^−1^ was also observed along with the chest computerized tomography (CT) scans revealing a reduction in the pulmonary lesions. The study reported no severe unpropitious effects, and suggested that CP therapy could possibly ameliorate the clinical outcomes by neutralizing viraemia in patients with critical SARS-CoV-2 infection and can serve as a promising rescue option for severe cases of COVID-19 [54].

Another study involved five patients, 36–65 years of age, of which two were female. The prime objective of the study was to determine whether the transfusion therapy of the CP was advantageous for the severely sick patients of SARS-CoV-2 infection, who were suffering from critical respiratory ailments. The patients were subjected to transfusion with CP, which consisted of a SARS-CoV-2-specific antibody (i.e. IgG). A neutralization titre greater than 40 (endpoint dilution titre) along with IgG-binding titre greater than 1:1000 (endpoint dilution titre), which was derived from five donors who successfully recovered from COVID-19 earlier, was administered to the patients between 10 and 22 days after they were admitted. During the treatment, all five patients were provided with mechanical ventilation along with methylprednisolone and other antiviral agents. The normalization of body temperatures was observed within 3 days in four of the five patients. The patients tested negative for SARS-CoV-2 within 12 days after CP transfusion, and the titres of neutralizing antibodies were also increased. The study concluded that the administration of CP consisting of the SARS-CoV-2-specific neutralizing antibodies in critically ill patients might have improved their clinical symptoms, and suggested that CP infusion could be a potential therapeutic strategy against COVID-19 [53], though the observations and results obtained from these studies need to be analysed and evaluated in further clinical trials.

## 7. Ongoing clinical trials

After the emergence of COVID-19 as a global pandemic, scientists across the world started looking for specific suitable treatments and prevention strategies. Around 69 studies are registered on clinicaltrials.gov that focus on dealing with the COVID-19 crisis with the help of CP therapy. The study entitled ‘Convalescent Plasma as Therapy for COVID-19 Severe SARS-CoV-2 Disease (CONCOVID Study)’ (clinical trial no. NCT04342182) is a randomized comparative trial which aims to evaluate the efficacy and safety of CP from COVID-recovered donors as a cure for symptomatic patients of COVID-19 in the hospital. In another study entitled ‘Early Transfusion of Convalescent Plasma in Elderly COVID-19 Patients to Prevent Disease Progression’ (clinical trial no. NCT04374526), the investigators hypothesize that the transfusion of CP, comprising the neutralizing antibodies, at an early stage of COVID-19 infection can prevent the inflammatory response induced by SARS-CoV-2. The main objectives of this trial include the prevention of pneumonia progression in elderly COVID-19 patients (more than 65 years of age), raising anti-SARS-CoV-2 antibody titres in recipients and decreasing the viral load using the CP therapy, along with standard therapy.

Moreover, a report titled ‘Convalescent Plasma Trial in COVID -19 Patients' (clinical trial no. NCT04356534) is also a randomized trial with an objective to compare plasma therapy using CP with an antibody against SARS-CoV-2 in COVID-19 patients with pneumonia and hypoxia and to find out whether there is any improvement in the clinical course. Another study called ‘Efficacy of Convalescent Plasma Therapy in Severely Sick COVID-19 Patients’ (clinical trial no. NCT04346446) is a randomized controlled trial, which focuses on assessing the efficacy of CP in COVID-19 patients. It aims to collect 500 ml CP from COVID-19-infected recuperated patients after 14 days of radiological and clinical recovery, with two subsequent negative PCR tests of COVID-19. It further aims to analyse the plasma sample for COVID-19-specific antibodies and their titres. Additionally, a study titled ‘Convalescent Plasma for Treatment of COVID-19: An Exploratory Dose Identifying Study’ (clinical trial no. NCT04384497) aims to treat a high-risk population that has viraemia before they develop any pulmonary infection/injury and start relying on supplementary oxygen therapy.

## 8. Conclusion

The infectivity and rate of transmission of SARS-CoV-2 necessitates the development of an effective and operational therapeutic approach as early as possible. The CP therapy approach for COVID-19 can be implemented as an immediate therapeutic to control the disease, in the absence of appropriate drugs or vaccine. Since CP therapy has been a prevalent and effective mode of treatment in various viral infections in the past (e.g. Ebola, MERS and SARS-CoV [84]), the WHO is considering it for the control of COVID-19 as well. CP therapy should be explored further to make it suitable and effective in the treatment of the patients infected with SARS-CoV-2.

In summary, the CP approach has already played an important role in the therapy of several viral diseases. In the case of COVID-19, to date, this is the only specific method that is able to encounter the SARS-CoV-2 antigen and hence can prove effective in saving the lives of people across the globe suffering from COVID-19.

## Data accessibility

This article has no additional data.
